# Effect of bone morphology of the tibia plateau on joint line convergence angle in medial open wedge high tibial osteotomy

**DOI:** 10.1186/s12891-022-05526-z

**Published:** 2022-06-13

**Authors:** Junya Itou, Umito Kuwashima, Masafumi Itoh, Ken Okazaki

**Affiliations:** Department of Orthopaedic Surgery, Tokyo Women’s Medical University, 8-1 Kawada-cho, Shinjuku-ku, Tokyo, 162-8666 Japan

**Keywords:** High tibial osteotomy, Joint line convergence angle, Bone morphology

## Abstract

**Background:**

Change in the joint line convergence angle (JLCA) of the knee after high tibial osteotomy (HTO) is difficult to predict accurately. Given that any change in JLCA is intra-articular, the shape of the articular surface, including the bone morphology of the proximal tibia, may affect the alignment of the knee joint postoperatively. The purpose of this study was to investigate the relationship between the shape of the tibial plateau and postoperative alignment of the knee joint by focusing on changes in JLCA.

**Methods:**

One hundred and nine knees that underwent HTO were retrospectively reviewed. The shape of the tibial plateau was classified based on the slope of the medial and lateral articular surfaces as depressed, flat (within 3 degrees), or convex (pagoda-like). The relationship between the shape of the tibial plateau and radiological parameters was investigated.

**Results:**

The shape of the tibial plateau was depressed in 38 knees, flat in 52 knees, and pagoda-like in 19 knees. There was a moderate correlation between the postoperative change in JLCA and the preoperative hip-knee-ankle angle for knees with a pagoda-shaped tibial plateau (*r* = 0.56) but not for the other two shapes.

**Conclusions:**

These findings suggest that knees with marked varus deformity before HTO are likely to show more change in JLCA postoperatively if the tibial plateau is pagoda-shaped than if it has a depressed or flat shape. The advantage of focusing on the bone morphology of the proximal tibia is that surgeons can easily perform visual assessment using preoperative radiograph.

## Introduction

Accurate alignment correction is necessary in high tibial osteotomy (HTO). However, it has been reported that only about 50–85% of knee joints are in target alignment after HTO [1–6]. Although many factors contribute to intraoperative correction errors [4, 7], change in joint line convergence angle (JLCA) is particularly difficult to predict, especially in patients with a large preoperative JLCA (e.g., > 3 degrees) [3, 8]. Overestimating the predicted change in JLCA would lead to undercorrection while underestimating it would lead to overcorrection. Therefore, it would be useful to have an indicator that can predict change in JLCA before and after surgery and ensure accurate correction.

Change in JLCA is related to several factors, including soft tissue laxity [8]. The shape of the tibial plateau is known to vary widely [9] and given that any change in JLCA is intra-articular, it is likely that change in JLCA might be affected by variation in the shape of the medial tibial plateau.

The purpose of this study was to determine the relationship between the shape of the tibial plateau before surgery and the alignment of the knee joint postoperatively, in particular change in JLCA. We hypothesized that the shape of the tibial plateau could potentially predict JLCA after surgery.

## Materials and methods

This retrospective study was approved by the institutional ethics committee of Tokyo Women’s Medical University (approval number: 4952) and included 109 knees (93 patients; 40 men, 53 women) with osteoarthritis (OA) or osteonecrosis (ON) that underwent HTO between January 2017 and December 2020 at our institution. Mean age was 56.4 (32–74) years. In all patients, both knees were assessed preoperatively and postoperatively on full-length weight-bearing radiographs.

The surgical procedures were performed by 4 specialist knee surgeons using a long locking plate (TriS, Olympus Terumo Biomaterials, Japan). HTO was performed by the open-wedge [10] or open-wedge distal tibial tuberosity osteotomy (OWDTO) method [11]. The target point at which the postoperative mechanical axis passes through 62.5% of the tibial plateau is set to the so-called Fujisawa point [12]. Open-wedge HTO was performed as follows. An oblique 7-cm longitudinal incision was made at the anteromedial proximal tibia. The pes anserinus and superficial medial collateral ligament were then carefully released from the tibia, whereas the deep medial collateral ligament above the osteotomy line was not detached. The starting point of the osteotomy was 35 mm distal from the medial tibial joint line, aiming towards the proximal third of the tibiofibular joint. Next, an ascending osteotomy was performed to complete the biplanar osteotomy. Artificial bone (OSferion 60, Olympus Terumo Biomaterials, Japan) was then inserted into the osteotomy gap. Finally, a TriS plate was fixed in place on the medial side of the tibia using 8 locking screws. The OWDTO procedure included triplane osteotomy using a TriS plate combined with an additional bicortical fixation from the tuberosity to the posterior tibia in the anterior-posterior direction and placement of artificial bone in the osteotomy gap, as previously described [11].

**Fig. 1:**
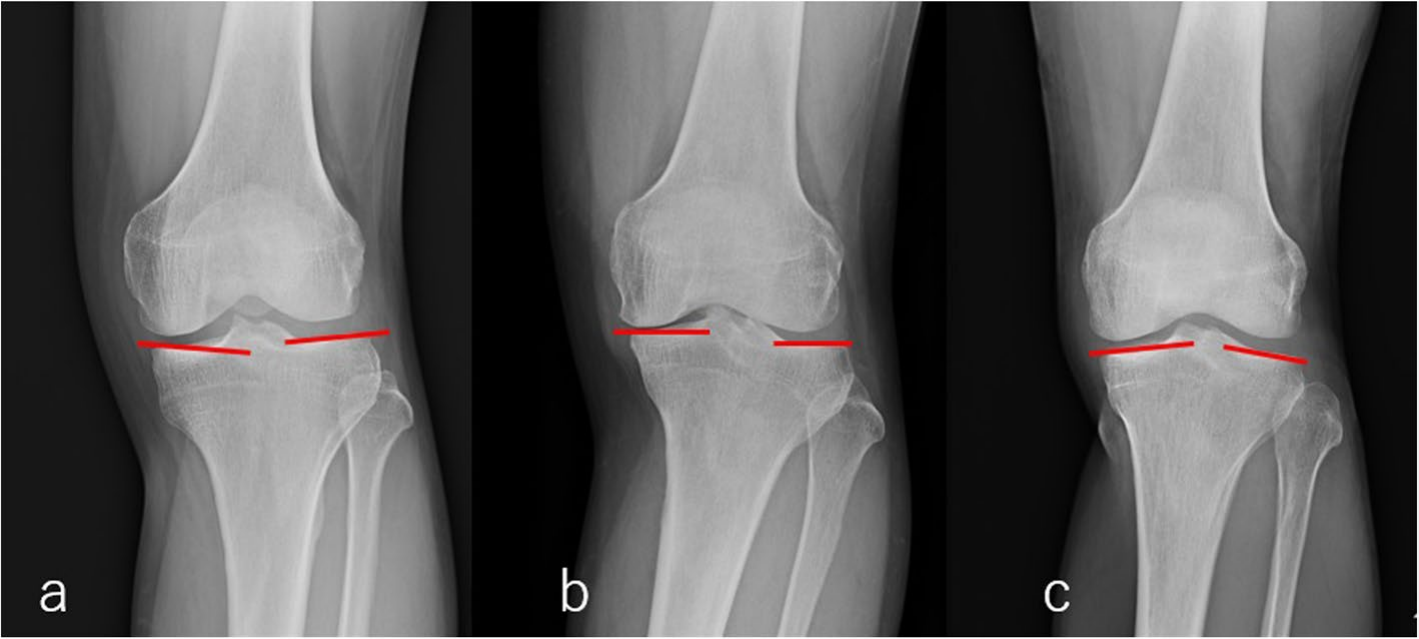
Shape of the tibial plateau. **a** Depressed. **b** Flat. **c** Convex (pagoda-like)

**Fig. 2:**
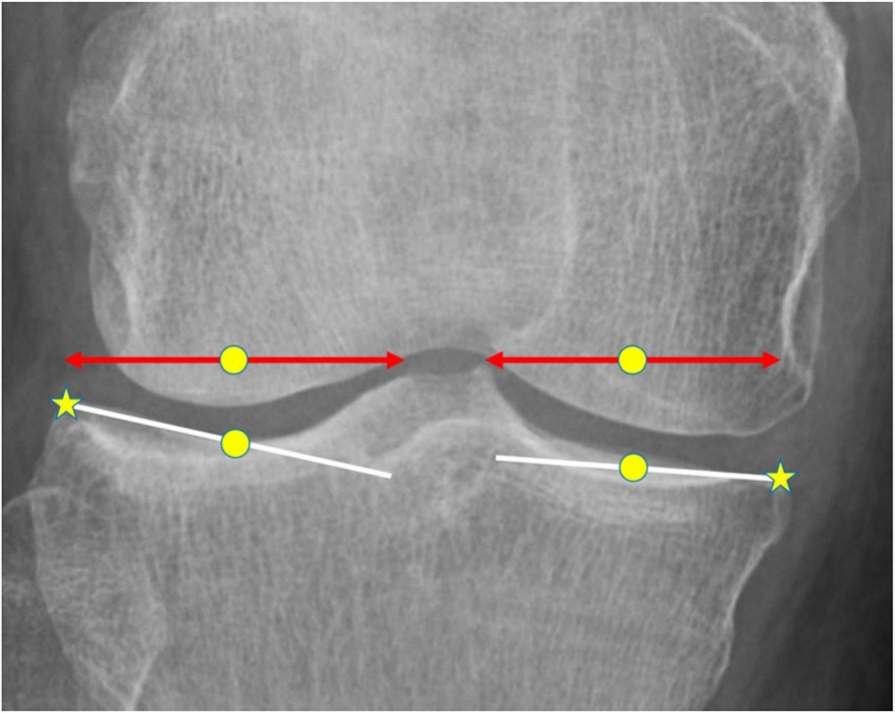
Definition of the slope (inclination) of the articular surface. The inclination was defined as the line connecting the tibial outer edge (star) and the midpoint between the tibial outer edge and the apex of the intercondylar ridge (circle and double arrows, respectively)

The shape of the tibial plateau was classified based on the slope of the medial and lateral articular surfaces as flat, depressed, or convex (pagoda-like) [9]. The shape was defined as flat if the slope of the medial and lateral articular surfaces measured within 3 degrees (Fig. 1). The shape of the tibial plateau was depressed in 38 knees, flat in 52 knees, and pagoda-like in 19 knees. The slope (inclination) was defined as the line connecting the tibial outer edge and the midpoint between the tibial outer edge and the apex of the intercondylar ridge (Fig. 2). The inclination of the articular surface was defined as positive when the angle was convex on top.

The radiological parameters measured included the medial proximal tibial angle (MPTA), hip-knee-ankle angle (HKA), mechanical lateral distal femoral angle (mLDFA), and JLCA. JLCA was defined as the angle between the tangent lines of the articular surfaces of the femur and tibia [8] and was taken to be positive when the intersection point was medial and negative when it was lateral. Patient data, including age, sex, preoperative body mass index, and preoperative and postoperative extension and flexion angles of the knee were collected from their medical records. Postoperative change in each radiological parameter was calculated as ΔMPTA, ΔHKA, and ΔJLCA [postoperative radiological parameter – preoperative radiological parameter] on full-length weightbearing radiographs. Correlations between the radiological parameters were analyzed according to the shape of the tibial plateau.

Data were analyzed using the Kruskal-Wallis test, chi-squared test, and one-way analysis of variance. Correlations were determined using a simple linear regression model. The intraobserver and interobserver reliability of each measurement were assessed using the intraclass correlation coefficient (ICC). Measurements were repeated after a 2-week interval to evaluate the intraobserver ICCs for the radiological parameters (MPTA, HKA, JLCA, and shape of the tibial plateau). In the assessment of intraobserver agreement, the ICCs for the radiological measurements of MPTA, HKA, JLCA, and shape of the tibial plateau were 0.89, 0.90, 0.94, and 0.95, respectively. In addition, for the interobserver reproducibility for the radiological parameters (HKA and shape of the tibial plateau), evaluations were performed individually by two observers (J.I and U.K.). The interobserver ICCs were 0.89, and 0.94, respectively.

Statistical analyses were performed using JMP software version 15 (SAS Institute Inc., Cary, NC, USA) and G*Power version 3.1.9.6 (Universität Kiel, Kiel, Germany). Post hoc analysis was performed to determine the statistical power of the correlation between ΔJLCA and radiographic parameters. With an effect size of 0.3, alpha of 0.05, and sample size of 109, the statistical power was 0.79. A *p*-value < 0.05 was considered statistically significant.

## Results

There was no significant difference in age, sex, or preoperative OA grade (without ON) or in the preoperative and postoperative radiological parameters measured (MPTA, HKA, mLDFA, and JLCA) according to the shape of the tibial plateau (Tables 1 and 2).

**Table 1:** Patient characteristics

	Depressed (*n* = 38)	Flat (*n* = 52)	Pagoda-like (*n* = 19)	*p*-value
Age (years), mean (SD)	57.2 (10.0)	56.5 (7.2)	54.5 (8.0)	0.53
Male sex, n (%)	13 (34.2)	23 (44.2)	12 (63.1)	0.11
Body mass index (%)	25.6 (3.9)	26.8 (4.0)	27.7 (4.0)	0.17
Preoperative knee extension angle (°)	1.1 ± 2.1	1.2 ± 2.5	1.0 ± 2.1	0.98
Preoperative knee flexion angle (°)	135.5 ± 9.5	138.2 ± 8.1	135.0 ± 8.3	0.30
K/L grade (1/2/3/4)	9/10/15/1	20/13/12/2	5/5/6/1	0.74
Procedure (OWHTO/OWDTO)	26/12	42/10	16/3	0.28

*K/L grade* Kellgren-Lawrence grade, *OWHTO* open-wedge high tibial osteotomy, *OWDTO* open-wedge distal tibial tuberosity osteotomy, *SD* standard deviation

The knee extension angle is expressed as 0° for full extension, with a negative value for hyperextension and as a positive value for limitation of extension

Data were analyzed using the Kruskal-Wallis test, chi-squared test, and one-way analysis of variance

**Table 2:** Radiographic data

	Depressed (*n* = 38)	Flat (*n* = 52)	Pagoda-like (*n* = 19)	*p*-value
MPTA (°) before surgery	83.8 (2.3)	83.8 (2.1)	83.6 (3.2)	0.93
MPTA (°) after surgery	91.7 (2.4)	91.4 (2.3)	91.9 (1.8)	0.71
HKA angle (°) before surgery	175.3 (2.5)	175.0 (1.8)	174.8 (2.5)	0.71
HKA angle (°) after surgery	183.6 (1.8)	183.4 (1.9)	183.3 (1.8)	0.78
JLCA (°) before surgery	2.2 (1.5)	2.3 (1.4)	2.5 (1.8)	0.82
JLCA (°) after surgery	1.6 (1.7)	1.6 (1.4)	1.5 (1.7)	0.92
mLDFA (°) before surgery	87.0 (1.6)	87.1 (1.3)	87.3 (2.1)	0.78
Inclination (°) before surgery	−7.4 (2.6)	0.1 (1.7)	6.0 (1.9)	< 0.01

*MPTA* medial proximal tibial angle, *HKA* hip-knee-ankle angle, *JLCA* joint line convergence angle, *mLDFA* mechanical lateral distal femoral angle

Data were analyzed using the Kruskal-Wallis test and one-way analysis of variance

**Fig. 3:**
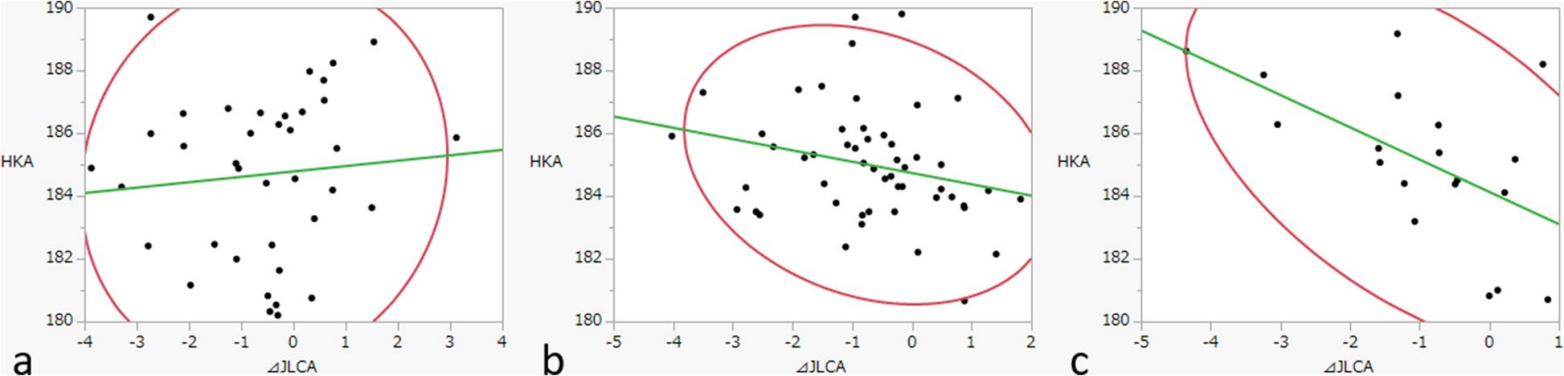
Correlations of ΔJLCA with HKA before surgery. **a** Depressed (*r* = − 0.09). **b** Flat (*r* = 0.24). **c** Convex (pagoda-like) (*r* = 0.56). ΔJLCA, change in joint line convergence angle of the knee after surgery; HKA, hip-knee-ankle angle

**Fig. 4:**
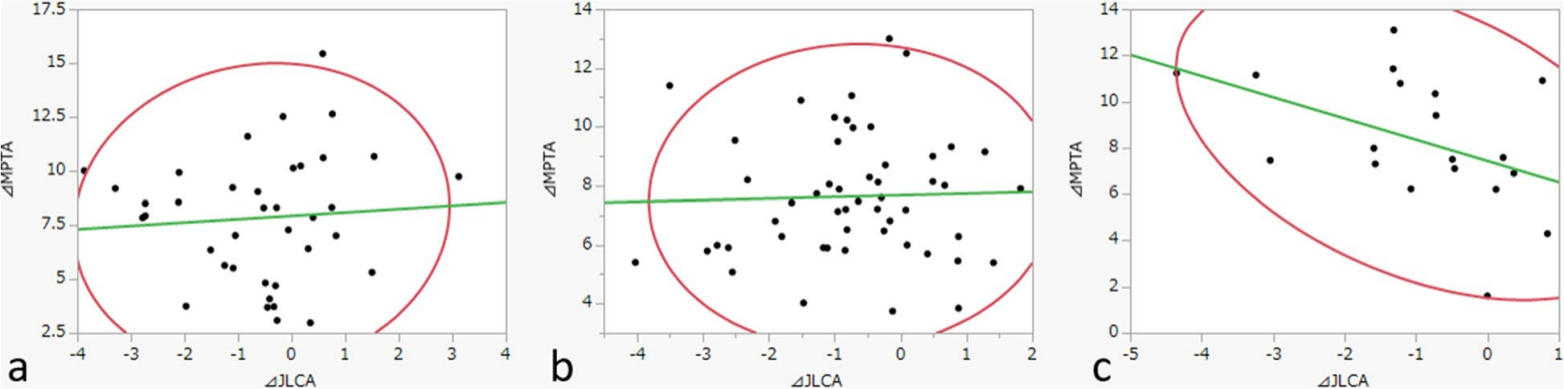
Correlations of ΔJLCA with ΔMPTA. **a** Depressed (*r* = 0.07). **b** Flat (*r* = 0.03). **c** Convex (pagoda-like) (*r* = − 0.44). ΔJLCA, change in joint line convergence angle of the knee after surgery; ΔMPTA, change in the medial proximal tibial angle

The ΔJLCA was moderately correlated with the preoperative HKA in knees with a pagoda-shaped tibial plateau (*r* = 0.56) but not in knees with the other two shapes (Fig. 3). Knees with a pagoda-shaped tibial plateau and marked varus deformity before surgery tended to have a greater ΔJLCA after surgery. Furthermore, there was a moderate correlation of ΔJLCA with ΔMPTA in knees with a pagoda-shaped tibial plateau (*r* = − 0.44) but not for the other two shapes (Fig. 4). Knees with a pagoda-shaped tibial plateau and a larger postoperative ΔMPTA tended to have a greater ΔJLCA. Regarding correlation analyses among various radiographic parameters, only knees with a pagoda-shaped tibial plateau showed a significant correlation with ΔJLCA, assuming changes between pre-operative and post-operative JLCA (Table 3).

**Table 3:** Results of correlation analyses among various radiographic parameters for knee joints with a pagoda-shaped tibial plateau

	JLCA (Pre)	JLCA (Post)	ΔJLCA	MPTA (Pre)	MPTA (Post)	ΔMPTA	HKA (Pre)	HKA (Post)	ΔHKA
JLCA (Pre)	1.00	0.69	−0.42	0.13	0.66	0.28	− 0.28	0.37	0.37
JLCA (Post)		1.00	0.35	0.35	0.52	−0.05	0.15	0.07	−0.06
ΔJLCA			1.00	0.27	−0.20	− 0.44	0.56	− 0.39	−0.57
MPTA (Pre)				1.00	0.48	−0.81	0.72	−0.34	− 0.64
MPTA (Post)					1.00	0.11	−0.04	0.35	0.20
ΔMPTA						1.00	−0.85	0.63	0.87
HKA (Pre)							1.00	−0.48	−0.90
HKA (Post)								1.00	0.81
ΔHKA									1.00

*MPTA* medial proximal tibial angle, *HKA* hip-knee-ankle angle, *JLCA* joint line convergence angle, *mLDFA* mechanical lateral distal femoral angle, *Pre* before surgery, *Post* after surgery

Δ represents the change in each radiological parameter after surgery

Correlations were determined using a simple linear regression model

## Discussion

To the best of our knowledge, this study is the first to investigate the relationship between JLCA and bone morphology of the proximal tibia and found some useful correlations, particularly for knees with a pagoda-shaped tibial plateau. The advantage of focusing on the bone morphology of the proximal tibia is that can be judged visually without the need for specific measurements. In this study, the most important finding was that knees with marked varus and a pagoda-shaped tibial plateau preoperatively had a larger change in JLCA.

An ability to predict the change in JLCA after surgery is important for obtaining accurate postoperative alignment [13]. In this study, knees with a pagoda-shaped tibial plateau and marked varus deformity preoperatively showed a greater change in JLCA after surgery that was correlated with the ΔMPTA, which is the amount of change in the correction angle. The difference in outcomes between knees with the pagoda-shaped tibial plateau and the other two shapes may be explained by the shear forces at the joint surfaces. In a study that included finite element analysis, Nakayama et al. [14] found that shear forces at the knee are generated by joint line obliquity. On the other hand, in a simulation study by Kuriyama et al. [15] the contact pressure between the femur and tibia changed with the changes in MPTA, but the shearing force did not differ significantly, indicating that there are many challenges remain. In another report, Hashemi et al. noted that the effect of coronal tibial slope on the biomechanics of the tibiofemoral joints is not fully understood [16]. However, it has recently been reported that a steep coronal tibial slope increases the load on the medial side of the tibiofemoral joint but not on the lateral side [17]. This finding is partially consistent with the report by Kuriyama et al. [15]. In the case of the pagoda-shaped tibial plateau, where the medial articular surface is strongly inclined, the contact pressure and shearing force applied to the tibiofemoral articular surface before and after osteotomy may differ between the medial and lateral sides. The pagoda-shaped tibial plateau has a tilted shape with medial and lateral notches, and the positional relationship between the femur and tibia may change in a seesaw pattern with movement of the load axis. In our study, when treating the pagoda-shaped knee joint by HTO, the ΔJLCA was predictable to some extent, so we were able to correct limb alignment accurately and achieve the desired clinical results. However, knees with the other shapes seemed to behave differently. In particular, depressed-shaped knees were more likely to lock because of their shape, without any significant change in JLCA even after a valgization HTO. However, the JLCA varies from case to case in clinical practice, which may be one of the reasons why JLCA has not been accurately predicted in past studies.

Even with careful surgical technique and preoperative planning, there is often a discrepancy between the alignment that is planned preoperatively and that achieved after surgery. This discrepancy has been attributed to laxity of soft tissue [4, 18], which is determined by the JLCA and thought to be the main cause of alignment correction error. Several studies have evaluated the relationship between JLCA and alignment correction parameters. Lee et al. [8] concluded that the preoperative JLCA correlates with lower limb alignment correction but not with correction error, which might reflect the fact that they did not analyze their data according to the bone morphology of the proximal tibia.

The results of the present study suggest that there was a proportional relationship between a greater postoperative change in JLCA and severe varus deformity before surgery in knees with a pagoda-shaped tibial plateau. This may be related to laxity of the lateral compartment, and the setting of the target alignment should be changed or another osteotomy method should be chosen, such as tibial condylar valgus osteotomy [9]. For knees with flat and depressed shapes, the behavior of the JLCA may be strongly influenced by soft tissue factors, including medial and lateral laxity. More evaluations of soft tissue that focus on the bone morphology of the tibial plateau are needed.

This study has several limitations. First, JLCA was assessed on only full-length weight-bearing radiographs and not on supine, varus stress, and valgus stress radiographs. So et al. [19] emphasized that the correction discrepancy after HTO was moderately correlated with the difference in JLCA between supine and standing radiographs. Nevertheless, in the present study, we focused on the ΔJLCA after surgery and found our JLCA measurements to be acceptable when obtained by a consistent method (i.e., full-length weightbearing radiographs). Second, there was a lack of definition regarding the bone morphology of the proximal tibia. For convenience, 3 degrees was used as a cutoff value to classify the three tibial plateau shapes. Further research is needed to determine what value is most appropriate and whether a new classification method is needed. Third, including knees with OA or ON as subjects was a disadvantage, given that there might be differences in subluxation of soft tissue between OA and ON, although a previous study [20] also included both OA and ON. All ON cases in our study were in the early stage without collapse of the femoral condyle, and thus there was no impact on the bone morphology of the femoral side. In addition, OA is generally diagnosed in knees with greater than Kellgren-Lawrence grade 2, although 34% of the knees in this study had Kellgren-Lawrence grade 1. However, previous studies have also included Kellgren-Lawrence grade 1 knees in their cohorts [3, 6]. Fourth, there were some cases with small correction in terms of ΔMPTA. In previous OWHTOs, the average correction angle was often 10 degrees or more [6, 19, 21]. However, the number of OWHTOs requiring ‘small’ corrections of less than 4 degrees has recently increased. In a study of OWHTO by Ogawa et al. [20], the minimal ΔMPTA was 3.3 degrees. The impact of these small corrections on JLCA was also considered a limitation of this study. Finally, surgical procedures that included release of the superficial medial collateral ligament (sMCL) may have affected the outcomes. However, Sato et al. [21] found no significant difference in laxity at 1 year after surgery even with sMCL release. More studies are needed to clarify the relationship between ΔJLCA and sMCL release.

## Conclusions

Although the ΔJLCA was to some extent predictable when HTO was performed in knees with a pagoda-shaped tibial plateau, careful preoperative planning may be necessary in cases with severe varus deformity and large correction angles. The advantage of focusing on the bone morphology of the proximal tibia is that surgeons can easily perform visual assessment using preoperative radiograph.

## Data Availability

The datasets used and/or analyzed during the present study are available from the corresponding author on reasonable request.

## Abbreviations

OWHTO: Open-wedge high tibial osteotomy
JLCA: Joint line convergence angle
OWDTO: Open-wedge distal tibial tuberosity osteotomy
MPTA: Medial proximal tibial angle
HKA: Hip-knee-ankle angle
mLDFA: Mechanical lateral distal femoral angle
